# Evaluating treatment modalities in chronic pain treatment by the multi-criteria decision making procedure

**DOI:** 10.1186/s12911-019-0925-6

**Published:** 2019-10-15

**Authors:** Ender Sir, Gül Didem Batur Sir

**Affiliations:** 1 Department of Algology and Pain Medicine, Gulhane Training and Research Hospital, Ankara, Turkey; 2 Department of Industrial Engineering, Gazi University, Ankara, Turkey

**Keywords:** Chronic pain treatment, Treatment selection, Multi-criteria decision making, AHP, TOPSIS

## Abstract

**Background:**

Chronic pain is one of the most common complaints of cancer patients. There are many pharmacological and non-pharmacological treatment modalities used for the treatment of pain. Nonetheless, non-pharmacological interventions are preferred because of potential side effects in cases resistant to medical therapy that require a dose increase or potent drug use. In most real-life situations, the decision on which technique to choose is based on the clinical but subjective decisions of the practitioners. This study aimed to find out the best non-pharmacological treatment option for patients with chronic cancer pain by following a rational and reasonable approach.

**Methods:**

Since the evaluation of treatment options requires to make a comparison between a number of alternatives in the light of certain criteria, we utilize the order relation analysis (G1-method) which is a method for determining the weights based on the improved Analytic Hierarchy Process (AHP). The method uses the relative importances on prioritizing the four criteria and eight sub-criteria defined by the experts of three pain physicians, one oncologist, and one oncologic surgeon. Four alternatives are then compared according to the Technique for Order Preference by Similarity to Ideal Solution (TOPSIS) using the verbal subjective judgments of the practitioners.

**Results:**

Obtained results indicate that the general medical condition of the patient and the stage of the cancer are the essential factors in the selection of the treatment method. It is followed by the extent of the pain and the level of evidence, respectively. According to the evaluations performed, spinal port and splanchnic nerve radiofrequency thermocoagulation treatments are the first and second priority methods for pain treatment, respectively, compared to lumbar epidural catheter and celiac plexus block.

**Conclusions:**

The results of this study emphasize the need to integrate critical criteria into the decision-making process objectively. This is the first study in which multi-criteria decision-making tools are used in the evaluation and selection of pain management methods in cancer patients.

## Background

Chronic pain is defined as persistent pain, which lasts longer than three months, and usually requires long-term treatment. It is a global health problem and is present in the range of one-third to 50 % of the population [1]. Chronic pain, which affects millions of people every year, is the most common cause of disability and is the most important cause for reducing the life quality. It is also problematic for the community, in terms of health costs and loss of productivity, for which the possible effects are higher than those of heart disease, diabetes, or cancer. The loss of working days related to chronic pain leads to economically dramatic losses due to expenditures in health units and compensations paid.

Cancer patients are among the most frequently concerned focus groups having chronic pain. Especially, upper abdominal malignancies lead to chronic, intractable pain for patients. They may experience pain due to direct tumor effects (e.g., metastatic bone invasion) or from adverse events of treatments or pain associated with comorbidities [2]. There are a variety of ways to treat such pain; ranging from pain medications to specialized treatment techniques and therapies. Although pharmacological therapies are applied at first, the use of interventional pain management techniques has become increasingly more involved in the treatment plans of patients suffering from chronic pain. The combination of pharmacological and non-pharmacological therapies is the most appropriate choice for the treatment of chronic pain, although medication-based therapies may be disadvantageous due to their potential effects such as causing various drug addictions. However, there are many non-pharmacological intervention options that we can classify as peripheral techniques, cognitive-behavioral techniques, and techniques other than these two methods (acupuncture, placebo, surgical treatment, nerve blocks). One of the biggest challenges that practitioners face is to decide which non-pharmacological intervention is to be selected for any particular patient.

Multi-criteria decision making (MCDM) provides the appropriate selection between a number of alternatives, in the light of specific criteria. Using MCDM techniques, it is possible to determine and sort the preferred alternatives. As the decisions to be made in health systems are of vital importance, it is not a rational approach to leave them to the subjective judgments of individuals. In the literature, there are studies regarding the multi-criteria selection and evaluation of possible diagnosis and treatment options of various diseases. Among the ones published in the last five years; Wagner et al. [3] tried to develop a framework in order to assess the value of the rare disease treatments, and organized the quantitative criteria of the framework into a hierarchical MCDM model. Ijabi et al. [4] proposed a method for evaluating and selecting the most suitable detoxification method, based on the Technique for Order Preference by Similarity to Ideal Solution (TOPSIS). Suner et al. [5] used the Analytic Hierarchy Process (AHP) together with the decision trees in order to construct a decision support tool for rectal cancer treatment. Hsu et al. [6] evaluated the choice of oral phosphodiesterase type 5 inhibitors for the treatment of erectile dysfunction by using AHP. Lopez and Gunasekaran [7] used fuzzy logic based Vise Kriterijumska Optimizacija I Kompromisno Resenje (VIKOR) method for evaluating H1N1 Influenza vaccination strategies. Balubaid and Basheikh [8] used AHP to select the most appropriate oral hypoglycemic agent for use among newly diagnosed patients with type 2 diabetes. Hancerliogullari et al. [9] used fuzzy AHP and fuzzy TOPSIS while evaluating anesthesia methods for a pediatric surgical procedure. Malekpoor et al. [10] used a TOPSIS based approach in order to prescribe an optimal dose plan for radiotherapy treatment. Ji et al. [11] proposed a fuzzy decision-making framework for treatment selection and applied the approach on the treatment selection problem of a particular patient with verruca plantaris. Eghbali-Zarch et al. [12] presented a computer-aided medical decision support tool using two MCDM methods, namely Fuzzy Step-wise Weight Assessment Ratio Analysis (SWARA) and Fuzzy Multi-Objective Optimization by a Ratio Analysis plus the Full Multiplicative Form (MULTIMOORA), for pharmacological therapy selection of Type 2 Diabetes.

In this study, we discussed how to make a selection among various treatment options that can be used in the treatment of chronic pain. In patients with cancer, it is known that pain occurs in the majority of cases and that millions of people complain about pain due to cancer every day. Therefore, in this study, non-pharmacological intervention methods used in cancer patients are compared to each other with respect to a number of criteria.

## Methods

### Defining the treatment alternatives

The aim of this study is to select the best treatment alternative among the following four non-pharmacological methods for the chronic pain observed in upper abdominal malignancy patients: administration of lumbar epidural catheter (Alternative 1), spinal port (Alt. 2), celiac plexus block (Alt. 3), and splanchnic nerve radiofrequency thermocoagulation (Alt. 4). The alternative treatment methods considered are determined by the help of the literature review together with the opinions of the pain medicine experts, who are three pain physicians, one oncologist, and one oncologic surgeon.

Epidural catheters are placed into the epidural space to inject local anesthetics and narcotics in order to provide regional analgesia. With an epidural catheter, the pain of patients can be relieved within minutes, but this relief ends when the effect of the given medication is over. The major advantage of this approach is the easy implantation of the catheter without a need for an imaging device such as fluoroscopy or computed tomography. However, the main complication of this technique is the infection that may occur in the catheter’s entry site and the epidural space. This technique is available for up to several weeks with frequent cleaning and dressing of the incision site.

Spinal port is a surgically implanted device which allows delivering medications to the intrathecal space. The port is placed under the skin on the anterior face of the chest, and the drug is delivered to the subarachnoid region with a catheter. The most significant advantage of the spinal port is that it is a closed system and thus can be used for years by reducing the risk of infection in most. The major disadvantages are the cost of the system and the need to be more precise in dose adjustment.

Celiac plexus block with neurolytic agents such as phenol or alcohol is an established treatment modality in upper gastrointestinal malignancies. Computed tomography guidance is usually needed for this approach. The success of block is strongly correlated with the spread of neurolytic drug in the celiac area [13]. The pain relief obtained from the neurolysis of the celiac ganglion is good to excellent for averaging several months. This pain-free term with only one intervention is the major superiority of the procedure. However, due to the extent of malignancy or growth in celiac lymph nodes, the anatomy of the celiac plexus may be impaired; therefore, access to the celiac ganglia becomes difficult, or the spread of the neurolytic agent may be insufficient [14].

Splanchnic nerve radiofrequency thermocoagulation can be administered in patients who have distorted anatomy of celiac ganglion because of advanced malignancy [15]. This technique has good results in pain relief with an improvement in the quality of life [16]. Another advantage of this technique is that, especially in comparison with the celiac plexus block, usually fluoroscopy guidance is sufficient instead of computed tomography. The need for special radiofrequency equipment and fluoroscope is the disadvantage of this approach.

### Specification of the evaluation criteria

**Fig. 1:**
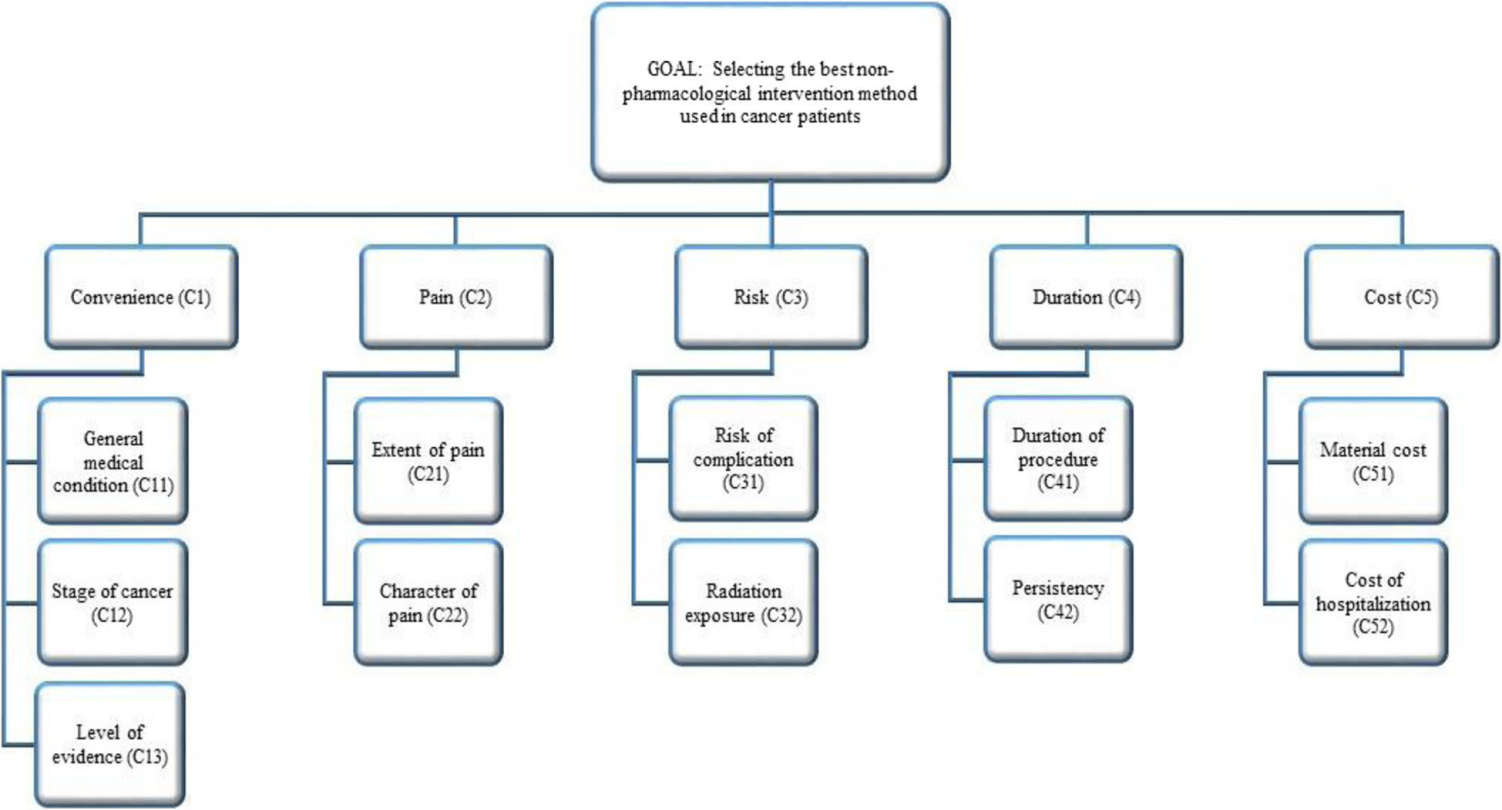
Hierarchical organization of the decision-making criteria

In the first part of this study, we want to determine the specific criteria that affect the choice of the treatment method. Thus, the initial step of the method was to create the problem hierarchy. At this point, the structure consisting of the main criteria and sub-criteria was defined. While determining the criteria, the literature regarding the choice of treatment method was examined, and the opinions of the experts related to the subject were consulted. In the light of the researches and evaluations, it is concluded that the choice of possible treatment should be done by considering the convenience (C1), pain (C2), risk (C3), duration (C4) and cost (C5) criteria. The hierarchical structure created for the problem is shown in Fig. 1.

The first criterion is convenience for which the subgroups are determined to be the patient’s general medical condition (C11), stage of cancer (C12), and level of evidence of the procedure (C13). For the assessment of the general medical condition, the medical history of the patient should be questioned, and a good physical examination should be performed. By this way, contraindications and risk of complications can be predetermined. The general medical condition is graded up according to the American Society of Anesthesiologists (ASA) physical status classification system. In this classification, ASA I and II are accepted as well, ASA III and IV as medium, and ASA V and VI as poor. The second one is the stage of cancer, which is evaluated with the “tumor, node, metastasis” (TNM) system. TNM is based on three main parameters, which are the extent of the tumor, spread to nearby lymph nodes, and spread to distant parts of the body (metastasis). Tumors without metastasis and does not spread to lymph nodes are low stage. Tumors which are spread to lymph nodes, without metastasis are intermediate stage. Tumors which are spread to lymph nodes and have metastasis are considered to be high stage, and almost all of these patients are inoperable and have low survival times. The last sub-criterion of this group is the level of evidence of the interventions for the individual diagnosis, that is obtained from well-designed studies, especially randomized controlled trials. It is indicated by a numerical value and a letter, followed by a positive or negative sign. 1A+, 1B+ and 2B+ indicates the highest, 2A+, 2B ∓ and 2C+ medium, and 0, 2C- and 2B- poorest recommendation levels. Consequently, the level of evidence indicates the recommendation rate of the procedure in light of the results from previous studies [17].

The second group of sub-criteria is defined under the heading of pain. The first one is the extent of the pain (C21), defining whether it is localized or extensive. The pain that only localized to the abdominal region is referred as well, having back pain in addition to abdominal pain as medium. Apart from these, it is accepted that pain, which includes distant areas such as widespread bone pain, shoulder pain, is ranked as poor. The second one is concerned with the character of pain (C22); which can be visceral, somatic, or neuropathic. Visceral pain is observed due to the tumor’s press on nerves, bones, or organs. Somatic pain occurs when cancer spreads to surrounding tissues or distant sites such as bone metastasis. Neuropathic pain is due to nerve damage caused by tumor pressing, radiotherapy, or chemotherapy. Having visceral pain is considered to be good, whereas visceral and somatic pain coexistence is considered as moderate, and visceral, somatic, and neuropathic pain observed at the same time is accepted as poor.

The third criterion title is the risk and its subheadings are the risk of complication of the procedure (C31) and the radiation exposure (C32). Each procedure has an individual risk of side effects, and complications vary from low to high. The complication risks of the procedures are evaluated according to the previous studies and the experience of the practitioners. For example, in the celiac plexus block, there are serious complications such as aortic and kidney injuries, while the epidural catheter has a low risk of dural puncture. Related to these possible risks of each of the procedures, the “benefit per complication” rate should be well evaluated before the procedure. Procedures with less risk of complications or have easy to manage complications are preferred to be applied first. Furthermore, there is a specific level of radiation exposure observed for some of the treatment options. Although the effects of radiation exposure are not immediately observed, it should be considered as a risk for long term complications. There is more radiation exposure in computed tomography (CT) guided procedures than fluoroscopy guided ones. While the spinal port and epidural catheter procedures do not need radiological guidance, the celiac plexus block usually administered under CT guidance and splanchnic radiofrequency is administered under fluoroscopic guidance.

Duration is the fourth criterion, and the sub-groups are the duration (C41) and the persistency of the procedure (C42). The four procedures performed in the study are administered under sedation without general anesthesia. In upper abdominal cancer patients, procedures performed in the shortest possible time should be selected as these patients cannot tolerate long-term procedures due to the pain that is aggravated with the position. The estimated duration of the procedures varies from a few minutes to more than an hour. We accept the duration of less than 10 min as short, and more than 30 min as the long ranked. The persistency of the treatment is another important outcome of the treatment in terms of patient comfort, causing no need to repeat the procedure. Persistency of a procedure can be a few days to a couple of years. Less than one month is accepted as short, and longer than three months is accepted as long term persistence.

The last criterion is cost, for which two sub-criteria are defined: the cost of the materials used (C51) and the cost of hospitalization (C52). Even though the management of pain and the clinical condition of the patients are the most important parameters, the financial aspect of the procedure is also important. According to the cost of materials used in the alternative treatments considered in our study, there is a huge difference between an epidural catheter and a spinal port. Thus, expensive systems are possibly preferred in patients who have long survival probabilities. In each procedure, different materials are used. We accepted less than fifty dollars as low, and higher than 500 dollars as high material cost. For last, factors affecting the duration of hospitalization are considered. Complications such as infection, bleeding, a patient’s ability to continue home treatment after the intervention, and the need for new procedures affect the length of hospital stay and increase the associated cost. The cost of hospitalization is related to the number of hospitalization days and the need for intensive care. In this study, outpatient procedures are accepted as short, hospitalization period up to 4 days as medium, more than 4 days or need of intensive care is considered as long length of stay.

### Determination of the weights of criteria

After defining the important criteria for the goal of selecting the best treatment alternative among the four non-pharmacological methods for the chronic pain observed in upper abdominal malignancy patients; we find out which one(s) among these criteria are more important over this decision, and determine which treatment method suits best to this specific group of patients. In this study, we propose a two-stage solution approach, in which we first determine the importance levels, namely the weights, of the sub-criteria that are important with respect to the selection and ranking problem that we focus on, and then we compare the alternatives with respect to the specific criteria and obtained weights, using the scorings of experts. While defining the solution procedure, the results presented by Wątróbski et al. [18] are used. Wątróbski et al. [18] presented the rules of selecting the most suitable MCDM method for a specific problem taking the basic descriptors of the decision problem into account. They decided on the method according to the scale on which the criterial performances are compared, the characterization of uncertainty, and the decision problematic. In our problem, we take different weights for the criteria into account which are relatively determined, while the alternatives are compared on a quantitative scale. The decision problem of ranking the alternatives and selecting the best one is assumed to have no uncertainty. Having these descriptors gives us the subset of AHP + TOPSIS as the most suitable approach.

With these aims of study, in the first stage we use the relation analysis method (G1-method) which is a simple and practical method for determining the weights based on the improved AHP method. Following this stage, the alternatives are ranked using another MCDM approach, namely TOPSIS method.

In the first stage of the procedure, we obtain the criteria weights using the scale value determined by the relative importance. The specific steps of the G1-method are as follows [19]:

*Step 1:* Determine the order relationship of the criteria.

The decision makers rank the criteria *C*_(1)_, *C*_(2)_, …, *C*_(*n* − 1)_, *C*_(*n*)_, which is determined as *C*_(1)_ ≻ *C*_(2)_ ≻ … ≻ *C*_(*n* − 1)_ ≻ *C*_(*n*)_ according to their importance, where *C*_(*i*)_ ≻ *C*_(*j*)_ indicates the importance degree of evaluation criterion *C*_(*i*)_ is greater (or not less) than *C*_(*j*)_.

*Step 2:* Determine the importance scale.

The relative importance of the evaluation criterion *C*_(*j* − 1)_ and *C*_(*j*)_ is defined by *r*_(*j*)_:

1 $$ {r}_{(j)}={w}_{\left(j-1\right)}/{w}_{(j)} $$

where *w*_(*j*)_, *w*_(*j* − 1)_ are the weights of the criteria (*j*) and (*j* − 1) respectively. However, *w*_(*j*)_ and *w*_(*j* − 1)_ are often unknown, but *r*_(*j*)_ can be obtained according to the importance degree of the two criteria by the decision makers. The values are shown in Table 1 [20].

**Table 1:** The value of relative importance

***r***_***j***_	Description
1.0	*C*_*j* − 1_ is the same importance as *C*_*j*_
1.1	Between the same and slightly more important
1.2	*C*_*j* − 1_ is slightly more important than *C*_*j*_
1.3	Between slightly more and more important
1.4	*C*_*j* − 1_ is more important than *C*_*j*_
1.5	Between more and strongly more important
1.6	*C*_*j* − 1_ is strongly more important than *C*_*j*_
1.7	Between strongly more and extremely more important
1.8	*C*_*j* − 1_ is extremely more important than *C*_*j*_

*Step 3:* Calculate the subjective weights *ω*_*sj*_ of each criterion by Eqs. (2)–(3)

2 $$ {\omega}_{sn}={\left[1+{\sum}_{i=2}^n\left({\prod}_{j=1}^n{r}_j\right)\right]}^{-1} $$

3 $$ {\omega}_{sj}={\prod}_{k=j+1}^n{r}_k{\omega}_{sn} $$

### The ranking and selection process

Once the weights are obtained, it is time to rank the alternatives and determine the best possible one. According to the TOPSIS technique, the best alternative is the one that is nearest to the positive-ideal solution which is the one maximizing the benefit criteria while minimizing the cost criteria, and farthest from the negative-ideal solution which maximizes the cost criteria while minimizing the benefit criteria. The method consists of the following steps [21]:

*Step 1:* Establish a decision matrix *D* for the ranking. This matrix can be shown as follows:

$$ D={\displaystyle \begin{array}{l}\kern0.5em {F}_1\kern0.5em {F}_2\kern0.5em \cdots \kern0.5em {F}_j\kern0.5em \cdots \kern0.5em {F}_n\\ {}\begin{array}{l}{\mathrm{A}}_1\\ {}{\mathrm{A}}_2\\ {}\vdots \\ {}{\mathrm{A}}_i\\ {}\vdots \\ {}{\mathrm{A}}_m\end{array}\left[\begin{array}{llllll}{f}_{11}& {f}_{12}& \cdots & {f}_{1j}& \cdots & {f}_{1n}\\ {}{f}_{21}& {f}_{22}& \cdots & {f}_{2j}& \cdots & {f}_{2n}\\ {}\vdots & \vdots & \cdots & \vdots & \cdots & \vdots \\ {}{f}_{i1}& {f}_{i2}& \cdots & {f}_{ij}& \cdots & {f}_{in}\\ {}\vdots & \vdots & \cdots & \vdots & \cdots & \vdots \\ {}{f}_{m1}& {f}_{m2}& \cdots & {f}_{mj}& \cdots & {f}_{mn}\end{array}\right]\end{array}} $$

where *A*_*i*_ denotes the alternatives *i*, *i* = 1, …, *m*; *F*_*j*_ represents *j*^*th*^ alternative or criterion, *j* = 1, …, *n*, related to *i*^*th*^ alternative; and *f*_*ij*_ is a crisp value indicating the performance rating of each alternative *A*_*i*_ with respect to each criterion *F*_*j*_.

*Step 2:* Calculate the normalized decision matrix *R*(=[*r*_*ij*_]). The normalized value *r*_*ij*_ is calculated by the following formula:

$$ {r}_{ij}=\frac{a_{ij}}{\sqrt{\sum_{j=1}^n{a}_{ij}^2}}\forall i,j $$

*Step 3:* Calculate the weighted normalized decision matrix by multiplying the normalized decision matrix by its associated weights. The weighted normalized value *v*_*ij*_ is calculated as:

$$ {v}_{ij}={w}_j\times {r}_{ij}\forall i,j $$

*Step 4:* Determine the positive-ideal and negative-ideal solutions, respectively:

$$ {V}^{+}=\left\{{v}_1^{+},\dots, {v}_n^{+}\right\}=\left\{\left(\underset{i}{\max }{v}_{ij}|j\in J\right),\left(\underset{i}{\min }{v}_{ij}|j\in J^{\prime}\right)\right\} $$

$$ {V}^{-}=\left\{{v}_1^{-},\dots, {v}_n^{-}\right\}=\left\{\left(\underset{i}{\min }{v}_{ij}|j\in J\right),\left(\underset{i}{\max }{v}_{ij}|j\in J^{\prime}\right)\right\} $$

where *J* is associated with the benefit criteria, and *J*′ is associated with the cost criteria.

*Step 5:* Calculate the separation measures, using the *m*-dimensional Euclidean distance. The separation measure ${D}_i^{+}$ of each alternative from the positive-ideal solutions is given as:

$$ {D}_i^{+}=\sqrt{\sum_{j=1}^n{\left({v}_{ij}-{v}_j^{+}\right)}^2},\forall i $$

Similarly, the separation measure ${D}_i^{-}$ of each alternative from the negative-ideal solution is as follows:

$$ {D}_i^{-}=\sqrt{\sum_{j=1}^n{\left({v}_{ij}-{v}_j^{-}\right)}^2},\forall i $$

*Step 6:* Calculate the relative closeness to the ideal solution and rank the alternatives in descending order. The relative closeness of the alternative *A*_*i*_ with respect to positive-ideal solution *V*^+^ can be expressed as:

$$ \overline{C_i}=\frac{D_i^{-}}{D_i^{+}+{D}_i^{-}},\forall i $$

where the index value $\overline{C_i}$ lies between 0 and 1. The larger the index value, the better the performance of the alternatives.

In order to obtain the decision matrix in the first step, experts are asked to make a criterion-based assessment for each alternative. In this step, according to the scores determined by the experts, each alternative is scored based on each of the eleven sub-criteria. The scoring table proposed, together with the experts, is given in Table 2. As can be seen from the table, scores are defined for each of the sub-criteria regarding to possible situations that can be observed under each subject. For example, patients having worse conditions like visceral, somatic, and neuropathic pain observed at the same time, or treatment modalities with low preferabilities due to high risk of complication, etc. are scored with higher values. Once the evaluations of the experts are obtained, the final scores are calculated taking the average score for each sub-criteria. Besides, in the third step of this algorithm, the weights obtained by the G1-method are used. Thus, the overall score for each alternative is obtained, and the best alternative, together with the final ranking among the alternatives are determined.

**Table 2:** The scores for the sub-criteria with respect to the alternatives

Criteria		C1		C2		C3		C4		C5	
Sub-criteria	C11	C12	C13	C21	C22	C31	C32	C41	C42	C51	C52
Score	Poor =3 Med. =2 Well =1	Early =1 Med =2 Late =3	Low = 1 Med = 2 High =3	Local =1 Med =2 Ext. =3	Visc. =1 Visc. + =2 Mikst =3	High =1 Med =2 Low =3	High =1 Med =2 Low =3	Long =1 Med =2 Short =3	Short =1 Med =2 Long =3	High =1 Med =2 Low =3	Long =1 Med =2 Short =3
Alt. 1	3	1	1	1	2	3	3	3	1	3	1
Alt. 2	2	3	2	3	3	2	3	1	3	1	1
Alt. 3	1	2	3	2	2	1	1	2	2,5	2,5	2
Alt. 4	1,5	2	2	2	2	1,5	2	1,5	2,5	2	2

## Results

The procedure given in “Methods” section is applied to the problem of selecting the best non-pharmacological intervention method used in cancer patients.

### The evaluation criteria and their weights

At the first step of the first stage, the criteria and the alternatives are defined, and the hierarchy of the criteria and alternatives is given in Fig. 1.

Then, the importance levels for each of the eight sub-criteria are determined. The comparisons are made by the evaluations of the four experts; 3 pain physicians (PP), 1 oncologic surgeon (OS), and 1 oncologist (OC). The experts determine the order of the sub-criteria and the rationale assignment of *r*_*j*_ values by referencing to Table 1. Related orders and rational assignments are given in Table 3. For example, second PP identified the general medical condition of the patient (C11) as the most important sub-criteria in determining the treatment modality, placing the stage of cancer (C13) as the second priority which he claimed to be of slightly less important than the other, having *r*_2_ value of 1.2. Obtained subjective priorities for the problem are given in Table 4. As can be seen from the table, general medical condition and stage of cancer have the highest priorities, and they are followed by localization and level of evidence, respectively.

**Table 3:** The orders of experts and rational assignments

Experts	Order	***r***_**2**_	***r***_**3**_	***r***_**4**_	***r***_**5**_	***r***_**6**_	***r***_**7**_	***r***_**8**_	***r***_**9**_	***r***_**10**_	***r***_**11**_
PP#1	*C*_11_ ≻ *C*_21_ ≻ *C*_12_ ≻ *C*_13_ ≻ *C*_22_ ≻ *C*_31_ ≻ *C*_42_ ≻ *C*_41_ ≻ *C*_32_ ≻ *C*_52_ ≻ *C*_51_	1, 0	1, 2	1, 4	1, 4	1, 2	1, 6	1, 4	1, 6	1, 8	1, 1
PP#2	*C*_11_ ≻ *C*_13_ ≻ *C*_31_ ≻ *C*_12_ ≻ *C*_21_ ≻ *C*_42_ ≻ *C*_22_ ≻ *C*_32_ ≻ *C*_41_ ≻ *C*_51_ ≻ *C*_52_	1, 2	1, 4	1, 4	1, 1	1, 4	1, 2	1, 6	1, 6	1, 8	1, 2
PP#3	*C*_11_ ≻ *C*_12_ ≻ *C*_21_ ≻ *C*_13_ ≻ *C*_31_ ≻ *C*_22_ ≻ *C*_41_ ≻ *C*_42_ ≻ *C*_32_ ≻ *C*_51_ ≻ *C*_52_	1, 4	1, 4	1, 5	1, 6	1, 6	1, 8	1, 2	1, 6	1, 8	1, 2
OS	*C*_12_ ≻ *C*_11_ ≻ *C*_13_ ≻ *C*_21_ ≻ *C*_31_ ≻ *C*_32_ ≻ *C*_42_ ≻ *C*_41_ ≻ *C*_22_ ≻ *C*_52_ ≻ *C*_51_	1, 6	1, 7	1, 5	1, 4	1, 6	1, 4	1, 4	1, 6	1, 4	1, 2
OC	*C*_11_ ≻ *C*_12_ ≻ *C*_21_ ≻ *C*_13_ ≻ *C*_42_ ≻ *C*_31_ ≻ *C*_22_ ≻ *C*_32_ ≻ *C*_41_ ≻ *C*_52_ ≻ *C*_51_	1, 4	1, 4	1, 7	1, 6	1, 4	1, 6	1, 5	1, 7	1, 2	1, 1

**Table 4:** Weights of the sub-criteria

Criteria	Weights
C1. Convenience	
C11. General medical condition	0,266
C12. Stage of cancer	0,221
C13. Level of evidence	0,134
C2. Pain	
C21. Localization	0,145
C22. Character of pain	0,046
C3. Risk	
C31. Risk of complication	0,079
C32. Radiation exposure	0,025
C4. Duration	
C41. Duration of procedure	0,021
C42. Persistency	0,045
C5. Cost	
C51. Material cost	0,009
C52. Cost of hospitalization	0,009

### Ranking and selection among alternatives

For the alternatives to be evaluated, scoring is performed, and criterion-based score values are obtained for each of the four alternatives, which are given in Table 2. These scores are then used by the TOPSIS algorithm together with the weight values determined in the previous stage, and the final score is found for each alternative. In order to obtain a more meaningful comparison, both the unweighted (when all of the sub-criteria are equally important) and the weighted rankings are presented in Table 5. According to the obtained results for the unweighted case, splanchnic nerve radiofrequency thermocoagulation is determined as the most preferred treatment alternative with the highest score. Spinal port, celiac plexus block, and epidural catheter are the second, third, and fourth choices, respectively. However, when we take the weights of the sub-criteria into account, meaning that some specific criteria are relatively more important in selecting the best treatment alternative, the spinal port becomes the first choice whereas it is followed by splanchnic nerve radiofrequency thermocoagulation. The third and fourth alternatives remain the same, where celiac plexus block is ranked to be the third and epidural catheter is the last. The main reason for this difference is that although splanchnic nerve radiofrequency thermocoagulation has satisfying scores for most of the criteria, it reaches relatively lower scores under localization and character of pain, which are the two important criteria for the selection problem.

**Table 5:** Best treatment alternative derived from the analysis

Alternative	Unweighted TOPSIS		Weighted TOPSIS	
Alt. 1	0,443	4th	0,205	4th
Alt. 2	0,591	2nd	0,800	1st
Alt. 3	0,550	3rd	0,498	3rd
Alt. 4	0,606	1st	0,746	2nd

## Discussion

It is one of the hardest decisions of physicians to give which treatment to which patient. The impact of personal judgments and preferences plays an important role at this point, where many independent criteria are effective in making such important decisions. These criteria often involve conflicting purposes. MCDM techniques stand out as a scientific approach commonly used at this point.

Throughout this study, evaluations from the experts are used together with the related literature research. At this point, it would be appropriate to explain the logic followed in defining the criteria-based scoring for alternatives. The patient group in which we try to determine the most appropriate treatment choice is the advanced cancer patients. For this group of patients, we determine a treatment method which has a high level of evidence, low complication risks, and costs. Since the criteria discussed have contradictory objectives, the evaluation is performed using MCDM logic. The techniques used in this process are determined according to the descriptors defined in the literature, and the results are obtained by the help of the opinions of experts concerned with pain treatment in cancer patients.

At the end of the study, spinal port treatment is found to be the most preferable treatment in upper abdominal cancer patients based on the opinions of experts participating in our study. It is a treatment modality which has been widely used in cancer patients to administer opioid intrathecally to provide a systemic effect. We know that intrathecal administration provides better analgesia with lower doses of opioids and local anesthetics [22]. However, when compared to other modalities, intrathecal analgesia via a spinal port has high invasiveness and cost. Therefore, this treatment is usually chosen at the last step. Actually, our results indicate that the choice between the implantable spinal port and the others depends primarily on the patient’s life expectancy. In patients with long life expectancy, although expensive and invasive, the spinal port becomes preferable. It is expected that the preferences of the experts become different from each other. However, when the rankings and preferences obtained in the final stage are presented, they also confirmed the logic of the results.

Our study also has some specific limitations. Decision-makers hesitated at some points in the evaluation of criteria and alternatives, and have difficulty in specifying exact values. At this point, the preference of using fuzzy or hesitant fuzzy term sets, which are frequently used in MCDM studies, may help decision-makers to be more comfortable in presenting their subjective judgments. Furthermore; in this study, a small number of experts working in a particular region could be consulted, and doctor-patient evaluations are not included. For the solution, in addition to these involvements, it is envisaged that a more accurate assessment can be made when a decision-making approach is used in which a larger number of experts is consulted and/or the preferences of the patients (or their relatives) are also involved. In the scope of this study we are mainly interested in the decision-making process, and the evaluation of the found treatments (compared to other possible treatments) is not focused on. However, any researcher can examine this issue, performing additional clinical studies. As it is mentioned before, this is a first study, hence the quality of the decision making process can be increased in follow-up studies in the hope that our manuscript is of enough interest.

## Conclusions

To the best of the authors’ knowledge, this is the first study focusing on the choice of treatment options for chronic pain. The problem addressed is a complex decision problem because each of the options discussed has its own advantages and disadvantages. In this study, we presented a G1 Method + TOPSIS based solution method for selecting the most suitable MCDM method for a specific problem. A number of effective criteria that are important in the treatment of chronic pain in cancer patients are defined by the help of the literature researches and the information obtained from the experts, and the problem is organized in a hierarchical framework consisting of five main criteria and eleven sub-criteria.

Different decision-making methods can be used for future research, and the results can be compared with the results obtained in this paper. It is possible to apply the proposed model to other multi-criteria decision-making problems in medical or health care procedures.

## Data Availability

The datasets used and/or analysed during the current study are available from the corresponding author on reasonable request.

## Abbreviations

AHP: Analytic Hierarchy Process
ASA: American Society of Anesthesiologists
CT: Computed tomography
MCDM: Multi-criteria decision making
MULTIMOORA: Multi-Objective Optimization by a Ratio Analysis plus the Full Multiplicative Form
OC: Oncologist
OS: Oncologic surgeon
PP: Pain physician
SWARA: Step-wise Weight Assessment Ratio Analysis
TNM: Tumor, Node, Metastasis
TOPSIS: Technique for Order Preference by Similarity to Ideal Solution
VIKOR: Vise Kriterijumska Optimizacija I Kompromisno Resenje
